# Systemic Sclerosis-Specific Determinants of Major Adverse Cardiovascular Events: Development of an Exploratory Cardiovascular Risk Model Beyond Traditional Risk Factors

**DOI:** 10.3390/diagnostics16193070

**Published:** 2026-09-22

**Authors:** Maria Laura Groseanu, Vlad Alin Rusu

**Affiliations:** 1 Faculty of General Medicine, Carol Davila University of Medicine and Pharmacy, Bld Eroii Sanitari nr 8, 050474 Bucharest, Romania; vlad-alin.rusu0720@stud.umfcd.ro; 2 Sfanta Maria Clinical Hospital, Bld Ion Mihalache 37-39, 011172 Bucharest, Romania

**Keywords:** systemic sclerosis, cardiovascular risk, major adverse cardiovascular events, pulmonary hypertension, cardiovascular prediction model

## Abstract

**Background**: Patients with systemic sclerosis (SSc) have an increased burden of cardiovascular disease, resulting from the combined effects of traditional cardiovascular risk factors, chronic inflammation, endothelial dysfunction, and disease-specific vascular involvement. Current cardiovascular risk prediction tools may not adequately capture the complex pathophysiological mechanisms contributing to cardiovascular complications in SSc. **Objectives**: This study aimed to evaluate the association between traditional cardiovascular risk factors and SSc-specific clinical characteristics with major adverse cardiovascular events (MACE) and to assess the incremental predictive value of disease-related variables beyond conventional cardiovascular risk factors. **Methods**: We conducted a retrospective observational cohort study including 272 SSc patients followed in a tertiary rheumatology center. The primary outcome was extended MACE, defined as the occurrence of cardiovascular death, myocardial infarction, ischemic stroke, hospitalization for heart failure, life-threatening arrhythmias, coronary or peripheral revascularization, and other major cardiovascular events. Logistic regression models were used to identify independent predictors of MACE. The incremental predictive value of SSc-specific variables was assessed by comparing model discrimination using receiver operating characteristic (ROC) curves, area under the curve (AUC), and Nagelkerke R^2^. **Results**: During a median follow-up period of 36 months (IQR 24–72), 77 patients (28.3%) experienced at least one extended MACE, while 21 patients (7.8%) fulfilled the restrictive definition of primary MACE. In univariable analysis, traditional cardiovascular factors associated with MACE included age at diagnosis (OR 1.03, 95% CI 1.01–1.05; *p* = 0.004), arterial hypertension (OR 2.33, 95% CI 1.35–4.00; *p* = 0.002), and dyslipidemia requiring statin therapy (OR 3.19, 95% CI 1.73–5.87; *p* < 0.001). Among SSc-specific variables, pulmonary hypertension showed the strongest association with MACE (OR 8.78, 95% CI 4.54–16.72; *p* < 0.001), followed by scleroderma renal crisis (OR 3.98, 95% CI 1.74–9.13; *p* = 0.001), digital ulcers (OR 3.71, 95% CI 1.94–7.07; *p* < 0.001), and interstitial lung disease (OR 3.43, 95% CI 1.80–6.53; *p* < 0.001). A conventional cardiovascular risk model demonstrated moderate predictive performance (Nagelkerke R^2^ = 0.149; AUC = 0.708). The sequential addition of SSc-specific variables resulted in a progressive improvement in model performance, with pulmonary hypertension producing the greatest incremental benefit (AUC = 0.811; Nagelkerke R^2^ = 0.326). An exploratory SSc-specific cardiovascular risk model including dyslipidemia treated with statins, diffuse cutaneous SSc, pulmonary hypertension, and C-reactive protein levels showed superior predictive performance (Nagelkerke R^2^ = 0.387; AUC = 0.821). **Conclusions**: Cardiovascular risk in SSc is not fully explained by traditional cardiovascular risk factors. Disease-specific characteristics, particularly pulmonary hypertension, diffuse cutaneous involvement, and inflammatory activity, provide significant incremental prognostic information. The integration of SSc-related variables into cardiovascular risk assessment models may improve identification of patients at increased risk of major cardiovascular events. The proposed model remains exploratory and requires external validation before clinical implementation.

## 1. Introduction

SSc is a rare, multisystem autoimmune connective tissue disease characterized by immune dysregulation, endothelial dysfunction, vasculopathy, and progressive fibrosis affecting the skin and internal organs [1]. Endothelial dysfunction is considered an early and central event in systemic sclerosis pathogenesis. Persistent endothelial injury promotes impaired angiogenesis, abnormal vascular remodeling, oxidative stress, and activation of inflammatory pathways, leading to progressive microvascular and macrovascular damage [2]. These processes may contribute to both peripheral manifestations, such as Raynaud phenomenon and digital ulcers, and internal organ complications involving the heart, lungs, and kidneys [3]. Furthermore, chronic autoimmune inflammation may accelerate atherosclerosis through mechanisms involving innate and adaptive immune activation, endothelial activation, and cytokine-mediated vascular damage [4]. Similar mechanisms have been described in other inflammatory rheumatic diseases, supporting the idea that systemic inflammation represents an independent cardiovascular risk enhancer [5].

Cardiovascular involvement in systemic sclerosis represents an important determinant of prognosis. Patients with SSc have an increased risk of cardiovascular events compared with the general population, including myocardial dysfunction, arrhythmias, pulmonary vascular disease, and accelerated atherosclerotic cardiovascular disease [6]. Although advances in diagnosis and treatment have improved overall survival, cardiovascular complications remain among the major determinants of morbidity and mortality in affected patients. Importantly, cardiovascular complications may occur through mechanisms that are not completely explained by traditional risk factors, suggesting a major contribution of systemic inflammation and disease-specific vascular injury [7].

Despite the recognized cardiovascular burden associated with SSc, validated cardiovascular risk prediction models specifically designed for this population are lacking. Most available risk assessment strategies rely on tools developed for the general population, without incorporating disease-specific characteristics that may substantially influence cardiovascular outcomes.

The primary aim of this study was to investigate the association between traditional cardiovascular risk factors, SSc-specific clinical characteristics, organ involvement, and inflammatory markers with major adverse cardiovascular events in a cohort of patients with SSc. The secondary aim was to evaluate whether the inclusion of SSc-specific variables improves the predictive performance of conventional cardiovascular risk models and to develop an exploratory SSc-specific cardiovascular risk model.

## 2. Materials and Methods

### 2.1. Study Design and Population

This retrospective observational cohort study included consecutive patients diagnosed with SSc who were followed in a tertiary rheumatology center (Sfanta Maria Clinical Hospital, Bucharest, Romania). Patients were identified from the institutional database and were included if they fulfilled the 2013 EULAR/ACR classification criteria for SSc and had available clinical, laboratory, imaging, and follow-up data required for cardiovascular outcome assessment.

The study was conducted according to the principles of the Declaration of Helsinki. The retrospective design and anonymized analysis of routinely collected clinical data were approved according to institutional ethical requirements. All participants provided informed consent for inclusion in the study and for the use of clinical and laboratory data for research purposes.

#### 2.1.1. Clinical Assessment and Collected Variables

The following demographic and disease-related variables were analyzed: sex, age at SSc diagnosis, age at inclusion, disease duration, SSc subtype, modified Rodnan Skin Score (mRSS), autoantibody profile, digital ulcers, nailfold videocapillaroscopy pattern (images obtained and interpreted by a single operator), specific organ involvement parameters, laboratory markers, and treatment.

Cardiac involvement was assessed through clinical evaluation, electrocardiography, echocardiography, laboratory markers, and medical records. Major cardiac involvement was defined as the presence of clinically relevant cardiac manifestations, including: heart failure, significant arrhythmias, ischemic cardiovascular events, and other major cardiac complications. NT-proBNP levels were analyzed as a biomarker of cardiovascular stress and myocardial involvement.

Pulmonary involvement was assessed using pulmonary function testing, thoracic imaging, and clinical parameters. The following pulmonary variables were analyzed: presence of ILD confirmed by computed tomography (CT), forced vital capacity (FVC, % predicted), diffusion capacity for carbon monoxide (DLCO, % predicted, six-minute walk distance (6MWD), and resting oxygen saturation. Interstitial lung disease (ILD) was defined based on specific imaging findings: peripheral and subpleural ground-glass opacities with lower-lobe and basilar predominance, accompanied by fine reticulation and traction bronchiectasis, architectural distortion, or honeycombing. Pulmonary hypertension (PH)was defined as a resting mean pulmonary arterial pressure (mPAP) greater than 25 mm Hg, measured via right heart catheterization.

Renal involvement evaluation included: serum creatinine, estimated glomerular filtration rate (eGFR), and history of scleroderma renal crisis. Renal crisis was defined as a documented diagnosis of acute renal involvement associated with SSc requiring appropriate medical intervention.

#### 2.1.2. Assessment of Traditional Cardiovascular Risk Factors

Traditional cardiovascular risk factors were assessed at baseline and included: male sex, age, smoking status, arterial hypertension, diabetes mellitus, and dyslipidemia. Hypertension was defined as a documented diagnosis of arterial hypertension or ongoing antihypertensive treatment. Diabetes mellitus was defined according to established diagnostic criteria (hemoglobin A1c ≥ 6.5% or fasting plasma glucose ≥ 126 mg/dL after fasting for at least 8 h or random plasma glucose: ≥200 mg/dL) or documented medical history. Dyslipidemia was defined based on previous diagnosis or treatment with lipid-lowering medication. Because lipid values may be modified by therapeutic intervention, the presence of dyslipidemia requiring statin therapy was used as a clinically relevant cardiovascular risk marker in predictive analyses.

### 2.2. Cardiovascular Outcomes

Because classic MACE definitions include only cardiovascular death, myocardial infarction, and ischemic stroke, which resulted in a limited number of events in this cohort, an extended MACE definition was used as the primary endpoint to increase statistical power. Extended MACE included: **(1)** coronary revascularization; **(2)** non-ST-segment elevation acute coronary syndrome (NSTEMI or unstable angina); **(3)** hospitalization for decompensated heart failure; **(4)** severe arrhythmias resulting in hemodynamic instability or resuscitated cardiac arrest; and **(5)** critical limb ischemia requiring peripheral revascularization.

The first cardiovascular event occurring during follow-up was considered for event classification.

### 2.3. Statistical Analysis

Continuous variables were assessed for distribution using appropriate normality tests and were presented as mean ± standard deviation (SD) or median with interquartile range (IQR), according to their distribution. Categorical variables were expressed as absolute numbers and percentages. Comparisons between groups were performed using Student’s *t*-test or Mann–Whitney *U* test for continuous variables, and chi-square test or Fisher’s exact test for categorical variables, as appropriate.

Univariable logistic regression analysis was performed to identify variables associated with the occurrence of extended MACE. Odds ratios (ORs), 95% confidence intervals (95% CIs), and *p*-values were calculated. Variables considered clinically relevant or showing significant association in univariable analysis were further evaluated in multivariable logistic regression models.

Multivariable logistic regression analyses were performed to identify independent predictors of cardiovascular abnormalities and major cardiovascular risk profiles in patients with SSc. Model calibration and discrimination were assessed using appropriate statistical methods, including goodness-of-fit testing and evaluation of the area under the receiver operating characteristic curve (AUC), when applicable.

All statistical analyses were performed using jamovi (version 2.7.31, The jamovi project).

Two predictive models were constructed.

The classical model included age at diagnosis, male sex, smoking status, arterial hypertension, diabetes mellitus, and dyslipidemia requiring statin therapy.

Based on univariable and multivariable analyses, an exploratory model was developed.

For survival analyses, when available, Kaplan–Meier curves were generated and differences between groups were assessed using the log-rank test. Cox proportional hazards regression models were used to evaluate independent predictors of cardiovascular events or mortality, with hazard ratios (HRs) and 95% CIs reported.

The incremental predictive value of SSc-specific variables was assessed by sequentially expanding the conventional cardiovascular model.

Model performance was evaluated using likelihood ratio chi-square (χ^2^), Nagelkerke R^2^, receiver operating characteristic (ROC) curve analysis, and area under the ROC curve (AUC).

The improvement in discrimination after adding SSc-specific variables was assessed by comparing AUC values between models.

## 3. Results

### 3.1. Baseline Characteristics of the Study Cohort

A total of 272 SSc patients were included in the study, predominantly females (86.8%), with a mean age of 53.3 ± 11.8 years at study inclusion and 50.4 ± 12.8 years at SSc diagnosis. The median disease duration was 4 years (IQR 2–10), while the median follow-up period of 36 months (IQR 24–72). The diffuse cutaneous SSc (dcSSc) and limited cutaneous SSc (lcSSc) were almost equally represented, whereas only five patients had sine scleroderma disease. Demographic data, disease characteristics related to organ involvement and cardiovascular risk factors are presented in Table 1.

### 3.2. Major Adverse Cardiovascular Events

During follow-up, 77 patients (28.3%) experienced at least one MACE according to the extended study definition. In all but two cases, the first cardiovascular event occurred after the diagnosis of SSc, supporting the temporal relationship between disease progression and cardiovascular complications. When the conventional three-point MACE was applied, only 21 patients (7.8%) fulfilled the endpoint, highlighting the relatively low frequency of traditional atherosclerotic cardiovascular events in this population (Table 2).

Hospitalization for decompensated heart failure represented the most frequent initial event (n = 35), accounting for nearly half of all first MACEs, followed by life-threatening arrhythmias or successfully resuscitated cardiac arrest.

### 3.3. Comparison Between Patients with and Without MACE

Patients who developed MACE during follow-up were older than those who remained event-free, both at disease onset and at study inclusion, whereas disease duration, follow-up duration, and sex distribution were comparable between groups (Table 3).

Among traditional cardiovascular risk factors, arterial hypertension and the use of lipid-lowering therapy were more frequent in patients who experienced MACE. Due to the greater use of lipid-lowering treatment, these patients had lower total and LDL cholesterol levels, while smoking status, diabetes mellitus, body mass index, HDL cholesterol, and triglyceride concentrations did not differ significantly between groups (Table 3).

Patients with MACE exhibited a more severe SSc phenotype. dcSSc, higher mRSS, advanced nailfold capillaroscopic abnormalities, digital ulcers, and a history of scleroderma renal crisis were all significantly more common in this group. Although ATA tended to be more frequent among patients with MACE, this association did not reach statistical significance, whereas RNA III-pol was observed only in patients without cardiovascular events.

Organ involvement was substantially more severe in patients who developed MACE. ILD was more prevalent, accompanied by poorer pulmonary function, reduced exercise capacity, lower resting oxygen saturation, and a markedly higher prevalence of PH, indicating more advanced cardiopulmonary involvement (Table 3).

Laboratory analyses revealed a more pronounced inflammatory and cardiovascular profile among patients with MACE. These patients exhibited higher inflammatory markers, including CRP and ESR, together with markedly elevated NT-proBNP concentrations, suggesting greater myocardial stress. They also demonstrated impaired renal function, reflected by higher serum creatinine levels and lower estimated glomerular filtration rate.

Regarding treatment, patients who experienced MACE were more frequently treated with glucocorticoids, immunosuppressive agents, and advance vasodilator therapy. No significant differences were observed in the use of conventional immunosuppressive agents, biologic therapies, or standard vasodilator treatment.

Overall, patients who developed MACE displayed a distinct clinical phenotype characterized by older age, diffuse cutaneous disease, advanced microvascular damage, more severe pulmonary and renal involvement, heightened systemic inflammation, and a greater burden of pulmonary hypertension. Collectively, these findings indicate that the occurrence of cardiovascular complications in systemic sclerosis is closely associated with overall disease severity and multisystem involvement, extending beyond the contribution of traditional cardiovascular risk factors.

### 3.4. Predictors of Major Adverse Cardiovascular Events

Univariable logistic regression identified several demographic, cardiovascular, disease-specific, and laboratory variables associated with the occurrence of extended MACE (Table 4). Among traditional cardiovascular risk factors, older age at SSc diagnosis, arterial hypertension, and dyslipidemia requiring statin therapy were significantly associated with cardiovascular events. In contrast, male sex, smoking status, and diabetes mellitus were not significantly associated with MACE.

Several disease-specific characteristics also emerged as significant predictors of cardiovascular events. Diffuse cutaneous SSc nearly doubled the odds of MACE, while mRSS, was associated with a modest but significant increase in cardiovascular. Patients with late capillaroscopic pattern were more than twice as likely to experience MACE, whereas the presence of digital ulcers and previous scleroderma renal crisis represented particularly strong markers of cardiovascular risk. ATA positivity showed a non-significant trend toward increased cardiovascular risk.

Pulmonary involvement demonstrated some of the strongest associations with cardiovascular outcomes. ILD was associated with a more than threefold increase in MACE risk. Among all evaluated variables, PH emerged as the single strongest predictor of MACE, conferring an almost ninefold increase in risk.

Higher CRP or ESR levels, NT-proBNP concentrations and serum creatinine levels were all significantly associated with MACE. Furthermore, patients receiving advanced vasodilator therapy exhibited a threefold higher likelihood of cardiovascular events, most likely reflecting the presence of more severe vasculopathy rather than a treatment-related effect.

To determine the independent contribution of traditional cardiovascular risk factors, a multivariable logistic regression model including age at diagnosis, sex, smoking status, arterial hypertension, diabetes mellitus, and statin-treated dyslipidemia was constructed (Table 5). The model was statistically significant (χ^2^ = 26.2, *p* < 0.001), explaining approximately 15% of the variance in cardiovascular events (Nagelkerke R^2^ = 0.149). After mutual adjustment, only statin-treated dyslipidemia remained independently associated with extended MACE, whereas age, hypertension, smoking, diabetes, and sex were no longer significant predictors.

### 3.5. Incremental Predictive Value of Systemic Sclerosis-Specific Variables

The baseline cardiovascular model, including only traditional cardiovascular risk factors, demonstrated moderate discrimination for predicting extended MACE, with an AUC of 0.708 and a Nagelkerke R^2^ of 0.149.

To evaluate whether disease-specific characteristics improve cardiovascular risk prediction beyond conventional cardiovascular risk factors, the baseline cardiovascular model was sequentially expanded by incorporating clinical, immunological, inflammatory, and organ-specific SSc variables (Table 6). Variables reflecting disease phenotype, including dcSSc, mRSS, ATA positivity, and late capillaroscopic pattern, produced modest but consistent improvements in predictive accuracy. Across these models, AUC values ranged from 0.721 to 0.731, while Nagelkerke R^2^ increased from 0.168 to 0.184, indicating that disease phenotype contributes additional prognostic information beyond conventional cardiovascular risk factors. The addition of ILD increased the AUC to 0.733 and the Nagelkerke R^2^ to 0.199. Likewise, the inclusion of CRP further enhanced model performance, increasing the AUC to 0.760 while nearly doubling the explained variance (Nagelkerke R^2^ = 0.267), supporting the contribution of systemic inflammation to cardiovascular risk in systemic sclerosis.

The model incorporating PH demonstrated the highest discriminatory ability among all extended models, with an AUC of 0.811 and a Nagelkerke R^2^ of 0.326.

Overall, these findings indicate that cardiovascular risk assessment in SSc is markedly improved by integrating disease-specific variables, particularly those reflecting pulmonary involvement and systemic inflammatory burden, rather than relying exclusively on traditional cardiovascular risk factors.

### 3.6. Development of an Exploratory Systemic Sclerosis-Specific Cardiovascular Risk Model

An exploratory multivariable logistic regression model was developed to identify a parsimonious combination of conventional cardiovascular and SSc-specific factors associated with extended MACE. Candidate variables included factors that showed a statistically significant association with extended MACE in the univariable analyses. Final variable selection was further guided by clinical and biological plausibility, with the aim of incorporating predictors representing complementary dimensions of cardiovascular risk and SSc-related disease burden.

Statin-treated dyslipidemia was selected as a conventional cardiovascular risk factor, while dcSSc was included as a readily identifiable disease phenotype established early in the clinical assessment of SSc. CRP was selected as a routinely available inflammatory biomarker reflecting systemic inflammatory burden and with potential relevance to cardiovascular risk. PH was included as an indicator of major cardiopulmonary organ involvement and disease severity and showed the strongest association with extended MACE in the univariable analysis. These variables were subsequently entered into the exploratory multivariable logistic regression model (Table 7).

Compared with the conventional cardiovascular model, the exploratory SSc-specific model demonstrated substantially improved predictive performance. The AUC increased from 0.708 to 0.821, while the explained variance increased from a Nagelkerke R^2^ of 0.149 to 0.387.

To evaluate the robustness of these findings, all analyses were repeated using the restrictive three-point MACE definition. Although the addition of PH and the exploratory variables increased the Nagelkerke R^2^, discrimination remained stable (AUC = 0.813), suggesting that the baseline cardiovascular model already performed well for classical cardiovascular events. Nevertheless, the consistent improvement observed after incorporating SSc-specific variables supports the robustness of the proposed model and indicates that these variables capture additional aspects of cardiovascular risk that are not adequately reflected by traditional cardiovascular risk factors alone.

## 4. Discussion

The present study demonstrates that cardiovascular risk in SSc reflects the complex interaction between conventional cardiovascular risk factors and disease-specific mechanisms of vascular injury. Although traditional determinants contributed to MACE, disease-related characteristics—particularly PH, dcSSc and systemic inflammation—provided substantial additional prognostic information. The sequential incorporation of SSc-specific variables progressively improved model discrimination, supporting the concept that cardiovascular involvement in SSc cannot be adequately captured by conventional cardiovascular risk assessment alone. Rather, cardiovascular disease should be considered a systemic manifestation of SSc-related vasculopathy, inflammation, and fibrosis.

Cardiovascular morbidity is increasingly recognized as an important component of the SSc disease burden [7]. Population-based studies have demonstrated increased risks of coronary artery disease and myocardial infarction in SSc, although the magnitude of risk varies according to the cardiovascular endpoint and population studied [8,9,10]. Importantly, cardiac involvement may occur in the absence of significant obstructive coronary disease. Microvascular dysfunction, recurrent myocardial ischemia, myocardial inflammation and fibrosis, and conduction abnormalities contribute to heart failure and arrhythmias and may precede clinically overt cardiac disease [11,12]. In our cohort, more than one quarter of patients experienced an event during follow-up when the extended MACE definition was applied, whereas fewer than 10% fulfilled the conventional three-point MACE endpoint. The predominance of heart-failure hospitalization as the initial event highlights the broader cardiovascular phenotype of SSc and supports the use of an extended endpoint that captures clinically relevant cardiac and pulmonary vascular complications.

Traditional cardiovascular risk factors nevertheless remained clinically relevant. Patients who developed MACE were older and more frequently had hypertension and statin-treated dyslipidemia. After adjustment, statin-treated dyslipidemia was the only conventional determinant independently associated with cardiovascular events. This finding should not be interpreted as evidence that other traditional risk factors are unimportant, particularly given the relatively low prevalence of diabetes and the limited statistical power for some exposures. Rather, treatment status may capture cumulative exposure to dyslipidemia more effectively than a single lipid measurement. The lower LDL cholesterol concentrations observed among patients receiving statins despite their higher event burden further illustrate the potential limitation of using current lipid values alone to estimate long-term cardiovascular risk. Similar discrepancies between lipid concentrations and cardiovascular risk have been described in chronic inflammatory diseases, in which inflammation may alter lipid metabolism and conventional risk estimates [5].

The moderate discrimination of the conventional model further emphasizes the limitations of general-population cardiovascular prediction tools in SSc. SCORE2, Framingham, and QRISK3 were developed primarily to estimate atherosclerotic cardiovascular risk and do not incorporate endothelial dysfunction, microvascular disease, pulmonary vascular involvement, myocardial fibrosis, or chronic immune activation [13]. Current EULAR recommendations acknowledge the increased cardiovascular burden across systemic rheumatic diseases but, because validated disease-specific tools remain unavailable for SSc, continue to recommend assessment using general-population cardiovascular prediction approaches together with careful management of traditional risk factors [14]. Our findings support the need for future SSc-specific models rather than suggesting that conventional risk assessment should be abandoned.

Disease-specific characteristics provided important additional prognostic information. Patients with MACE had a more severe phenotype, characterized by diffuse cutaneous involvement, digital ulcers, advanced microvascular abnormalities, pulmonary and renal involvement, increased inflammatory markers, and elevated NT-proBNP. dcSSc independently predicted MACE in the exploratory model, approximately doubling cardiovascular risk. This association is biologically plausible because diffuse disease is associated with greater systemic fibrosis, more frequent internal-organ involvement, and a higher burden of vascular injury. Recent data continue to demonstrate the close relationship between skin severity and cardiopulmonary disease, supporting the use of disease phenotype as an important component of risk stratification [15].

Digital ulcers and advanced nailfold capillaroscopic abnormalities also identified patients with a greater cardiovascular burden. Digital ulcers are increasingly regarded as manifestations of generalized SSc vasculopathy rather than isolated peripheral vascular disease. EUSTAR data have shown that a history of digital ulcers is associated with subsequent cardiovascular worsening and reduced survival [16]. Similarly, advanced capillaroscopic patterns have been associated with pulmonary hypertension, digital ulcers, disease progression, and shorter survival [17]. Importantly, recent multicenter evidence demonstrated that incorporation of nailfold capillaroscopy findings into SCORE2 significantly improved discrimination for cardiovascular events in SSc, providing independent support for the concept emerging from our study that microvascular assessment may add prognostic information beyond conventional risk factors [18].

Pulmonary and renal involvement showed similar associations with cardiovascular outcomes. Patients with MACE had more frequent ILD, lower FVC and DLCO, and shorter 6MWD. ILD increased cardiovascular risk in univariable analysis, although its effect was attenuated after adjustment for other disease-specific variables. This may indicate that ILD acts partly as a marker of overall disease severity and pulmonary vascular involvement rather than as an entirely independent cardiovascular determinant. Previous studies have similarly identified interstitial lung disease, reduced DLCO, and pulmonary vascular disease as important determinants of adverse outcomes in SSc [19,20,21]. Renal involvement, particularly previous scleroderma renal crisis, was also strongly associated with MACE in univariable analysis. Its loss of independent significance in the multivariable model likely reflects the close interrelationship between renal crisis, diffuse disease, PH, and systemic vascular injury rather than an absence of biological relevance.

Systemic inflammation represented another important component of cardiovascular risk. Patients with MACE had higher CRP and ESR, and CRP remained independently associated with cardiovascular events in the exploratory model. Chronic inflammation may contribute to cardiovascular injury through endothelial activation, oxidative stress, impaired nitric oxide bioavailability, leukocyte recruitment, vascular smooth-muscle proliferation, and extracellular matrix remodeling [22]. Recent SSc-specific evidence has demonstrated an association between persistent low-grade inflammation and increased arteriosclerotic cardiovascular risk, further supporting the relevance of CRP as more than a nonspecific marker of disease activity [23]. Our findings therefore reinforce the concept that systemic inflammation may function as a cardiovascular risk enhancer in SSc.

NT-proBNP was strongly associated with MACE, consistent with its ability to integrate myocardial and right ventricular stress, pulmonary vascular disease, and heart failure. NT-proBNP is already incorporated into contemporary PH screening and risk-assessment strategies, and elevated concentrations are associated with adverse outcomes in SSc [24]. In the present analysis, however, NT-proBNP was not included in the final exploratory prediction model because it may represent established cardiovascular involvement rather than a purely baseline risk determinant. Its strong association with events nevertheless supports its role in identifying patients requiring comprehensive cardiopulmonary assessment.

The most important finding was the dominant prognostic contribution of PH. PH was associated with an almost ninefold increase in the risk of MACE and produced the largest improvement in model performance, increasing the AUC from 0.708 for the conventional model to 0.811 after its incorporation. This finding is biologically plausible because PH represents the consequence of chronic endothelial injury, impaired angiogenesis, vascular proliferation, inflammation, and progressive vascular remodeling [25]. These mechanisms overlap with the systemic vasculopathy of SSc and may therefore identify patients with a particularly high burden of vascular injury across multiple vascular beds. Current ESC/ERS guidelines emphasize the high prevalence and prognostic importance of PH in SSc and recommend systematic multimodal screening using pulmonary function testing, echocardiography, and biomarkers such as NT-proBNP [24].

The prognostic importance of PH in our study extends previous observations that SSc-associated pulmonary arterial hypertension is a major determinant of mortality [25]. Importantly, our findings suggest that PH should not be viewed exclusively as a pulmonary complication. Rather, its presence may identify a broader cardiovascular phenotype characterized by myocardial dysfunction, right ventricular stress, arrhythmogenic substrate, and generalized vascular remodeling. Consequently, the identification of PH should prompt comprehensive cardiovascular assessment rather than evaluation restricted to the pulmonary circulation.

The progressive improvement observed after the sequential incorporation of disease-specific variables provides further support for this disease-centered interpretation. The exploratory model combining statin-treated dyslipidemia with diffuse cutaneous SSc, PH, and CRP achieved substantially better discrimination than the conventional model (AUC 0.821 vs. 0.708). Although these results require external validation, they suggest that combining conventional cardiovascular determinants with measures of systemic vasculopathy and inflammation may provide a more comprehensive assessment of cardiovascular vulnerability. This concept is consistent with emerging SSc research showing that disease-specific clinical, vascular, and imaging characteristics can improve cardiovascular prediction beyond conventional risk factors [18,26].

The clinical implications of our study are potentially important. Current cardiovascular assessment in SSc focuses predominantly on traditional risk-factor modification and surveillance for PH or myocardial involvement. Our findings support a more integrated approach, incorporating age and conventional cardiovascular risk factors together with disease phenotype, inflammatory activity, peripheral vascular manifestations, and pulmonary vascular involvement. Diffuse cutaneous disease, digital ulcers, CRP, capillaroscopic abnormalities, and particularly PH are readily identifiable during routine clinical evaluation and could help identify patients requiring intensified cardiovascular surveillance. Nevertheless, the present exploratory model should not yet be used for clinical decision-making until externally validated.

Several strengths should be acknowledged. This study included a well-characterized SSc cohort with a comprehensive assessment of traditional cardiovascular risk factors, disease manifestations, laboratory parameters, and cardiovascular outcomes. The extended MACE definition allowed a broader assessment of the cardiovascular phenotype of SSc, while sequential modeling quantified the incremental contribution of disease-specific variables. The identification of PH as the strongest predictor and the improvement obtained through incorporation of disease-related characteristics provide clinically relevant information for future risk-stratification research.

Several limitations must also be considered. The retrospective, single-center design limits causal inference and may have introduced residual confounding and referral bias. The cohort may therefore have included patients with more severe disease than the general SSc population. The extended MACE definition limits direct comparison with studies using conventional three-point MACE. Most importantly, the exploratory prediction model was internally derived and lacks external validation and a formal assessment of calibration; therefore, its findings should be considered hypothesis-generating. Given the use of an extended MACE definition, the model may capture heart failure hospitalizations and overall disease severity to a greater extent than classical atherosclerotic cardiovascular events. Finally, potentially informative biomarkers of endothelial activation and myocardial fibrosis, as well as advanced cardiovascular imaging parameters, were not systematically available and may further improve future models.

## 5. Conclusions

In conclusion, cardiovascular risk in SSc is determined by the interaction between conventional cardiovascular factors and disease-specific mechanisms of inflammation, endothelial dysfunction, vasculopathy, and fibrosis. Pulmonary hypertension emerged as the strongest predictor of cardiovascular events and provided the greatest incremental improvement in risk discrimination. The improved performance of a model incorporating statin-treated dyslipidemia, diffuse cutaneous disease, PH, and CRP supports the concept that cardiovascular risk assessment in SSc should move beyond traditional atherosclerotic risk factors toward an integrated, disease-specific approach. Prospective multicenter studies are now required to externally validate these findings, assess model calibration and clinical utility, and determine whether disease-adapted cardiovascular risk stratification can ultimately improve prevention and reduce cardiovascular morbidity and mortality in SSc.

## Figures and Tables

**Table 1:** Study cohort characteristics.

Category	Variable	Value
Demographic data	Female (n, %)	236 (86.8%)
	Age at diagnosis, years, (mean ± SD)	50.4 ± 12.8
	Age at inclusion, years, (mean ± SD)	53.3 ± 11.8
	Disease duration, years, (mean ± SD)	4 (2–10)
	Follow-up, months, (mean ± SD)	36 (24–72)
	Survival (n, %)	234 (86%)
Cardiovascular risk factors	Smoking (n, %)	75 (27.6%)
	Body mass index, kg/m^2^ (median, IQR)	22 (20–24)
	Arterial hypertension (n, %)	149 (44.4%)
	Diabetes mellitus (n, %)	13 (5%)
	Lipid-lowering treatment (n, %)	140 (55.3%)
	Cholesterol total, mg/dL (mean ± SD)	188 ± 45.7
	LDL, mg/dL, (median, IQR)	98 (71.8–129)
	HDL, mg/dL (median, IQR)	52 (42–62)
Disease characteristics	Disease subset (n, %)	
	Diffuse	134 (49.3%)
	Limited	133 (48.9%)
	Sine scleroderma	5 (1.8%)
	Autoantibodies (n, %)	
	ATA	152 (56.1%)
	ACA	94 (34.7%)
	RNA III-pol	12 (4.4%)
	CRP, mg/L, (median, IQR)	4 (1.6–8.4)
	ESR, mm/h (median, IQR)	17 (10–53)
	Nailfold capillaroscopy patterns	
	Early (n, %)	38 (14%)
	Active (n, %)	120 (44.1%)
	Late (%)	103 (37.9%)
	Non-scleroderma (n, %)	11 (4%)
	mRSS (median, IQR)	8 (5–12)
	ILD (n, %)	174 (64.3%)
	Mean FVC (%predicted ± SD)	83.1 ± 19.8
	Mean DLCO (%predicted ± SD)	67.1
	6MWD, m (mean ± SD)	445 ± 132
	PH (n, %)	57 (21.2%)
	mPAP, mmHg (median, IQR)	31.2 (22–33.5)
	Digital ulcers (n, %)	140 (55.1%)
	Scleroderma renal crisis (n, %)	9 (3.3%)
	Cardiac involvement	
	Arrhythmias (n, %)	103 (37.9%)36.9
	Heart failure (n, %)	79 (29.3)
	Pericardial disease (n, %)	34 (13)
	Nt-proBNP, pg/mL, (median, IQR)	282 (131–990)
SSc treatment	Corticosteroids (n, %)	119 (43.8%)
	Immunosuppressive medication (n, %)	225 (82.7%)
	Biologics (n, %)	22 (8.1%)
	Classic vasodilators (n, %)	203 (74.6%)
	Advanced vasodilators (n, %)	74 (27.2%)

SD—standard deviation; n—number; IQR—interquartile range; LDL—low-density lipoprotein; HDL—high-density lipoprotein; CRP—C reactive protein; ESR—erythrocyte sedimentation rate; ATA—antitopoisomerase 1 antibodies; ACA—anticentromer antibodies; RNA III-pol—antiRNA III polymerase antibodies; mRSS—modified Rodnan skin score; ILD—interstitial lung disease; FVC—forced vital capacity; DLCO—diffusing lung capacity of carbon monoxide; 6MWD—six-minute walking distance; PH—pulmonary hypertension; mPAP—mean pulmonary arterial pressure.

**Table 2:** Distribution of major cardiovascular events (MACE).

Event	n (%)
Primary MACE (n, %)	21 (7.8%)
Extended MACE (n, %)	77 (28.3%)
All MACE (n)	
Acute myocardial infarction	7
Ischemic or hemorrhagic stroke	5
Cardiovascular death	5
Coronary revascularization	3
Hospitalization for heart failure	35
Life-threatening arrhythmia or resuscitated cardiac arrest	11
Acute coronary syndrome without myocardial infarction	5
Critical limb ischemia or peripheral revascularization	5
Other cardiovascular events	2

**Table 3:** Comparison between SSc patients with MACE and without.

Category	Variables	Non-MACE (195)	MACE (77)	*p*
Demographic characteristics	Male sex (n, %)	21 (10.9%)	14 (18.2%)	0.110
	Age at diagnosis, years (mean ± SD)	48.9 ± 12.8	54 ± 12.3	0.003
	Age at inclusion, years (mean ± SD)	51.9 ± 11.6	56.6 ± 12.8	0.003
	Follow-up, months (mean ± SD)	36 (24–72)	48 (24–96)	0.088
	Disease duration, years (mean ± SD)	4 (2–9)	5 (3–11)	0.420
Cardiovascular risk factor	Smoking status (n, %)	50 (26%)	24 (31.2%)	0.395
	Arterial hypertension (n, %)	73 (38.4%)	45 (59.2%)	0.002
	Diabetes mellitus (n, %)	11 (5.9%)	2 (2.8%)	0.302
	Dyslipidemia + treatment (n, %)	86 (47.5%)	52 (74.3%)	<0.001
	Body mass index, kg/m^2^ (median, IQR)	22 (20–24)	22.5 (21–24)	0.732
	Cholesterol—total, mg/dL (median ± SD)	195 ± 43.3	167 ± 45.9	<0.001
	Cholesterol—HDL, mg/dL (mean ± SD)	53 (45–62)	47 (36–63)	0.172
	Cholesterol—LDL, mg/dL (mean ± SD)	103 (82.8–132)	84.5 (59.3–111)	0.005
	Triglycerids, mg/dL (mean ± SD)	91.5 (68.5–121)	88 (71.5–111)	0.872
SSc features	dcSSc (n, %)	85 (45.5%)	48 (62.3%)	0.013
	ACA (n, %)	72 (37.7%)	22 (28.6%)	0.157
	ATA (n, %)	100 (52.4%)	49 (63.6%)	0.093
	RNA III-pol (n, %)	12 (6.3%)	0 (0%)	0.024
	mRSS (median, IQR)	7 (5–12)	8 (6–14)	0.005
	NVC—late pattern (n, %)	62 (32.3%)	39 (50.6%)	0.005
	Digital ulcers (n, %)	88 (47.3%)	50 (76.9%)	<0.001
	ILD (n, %)	109 (56.8%)	63 (81.8%)	<0.001
	FVC %predicted (mean ± SD)	87.2 ± 18.6	73.5 ± 19.6	<0.001
	DLCO %predicted (mean ± SD)	69.6 (56.5–78)	56 (37.8–72.3)	<0.001
	PH (n, %)	19 (10.1%)	38 (49.4%)	<0.001
	Baseline 6MWD, m (mean ± SD)	464 ±125	405 ± 144	0.026
	Scleroderma renal crisis (n, %)	11 (5.7%)	15 (19.5%)	<0.001
Laboratory	CRP mg/L, (median, IQR)	3 (1–6.25)	6 (3–21)	<0.001
	ESR mm/h, (median, IQR)	14 (8.5–28.5)	33 (17–46)	<0.001
	NT-proBNP pg/mL (median, IQR)	146 (86.6–350)	863 (263–2773)	<0.001
	Creatinine mg/dL (median, IQR)	0.9 (0.8–1.01)	1 (0.84–1.22)	0.002
Treatment	Corticosteroids	77 (40.1%)	42 (54.5%)	0.03
	Immunosuppressive medication	155 (80.7%)	70 (90.9%)	0.041
	Biologics	14 (7.4%)	7 (9.1%)	0.644
	Classic vasodilators	151 (78.6%)	52 (67.5%)	0.056
	Advanced vasodilators	38 (19.8%)	34 (44.2%)	<0.001

SD—standard deviation; n—number; IQR—interquartile range; dcSSc—diffuse cutaneous involvement systemic sclerosis; LDL—low-density lipoprotein; HDL—high-density lipoprotein; CRP—C reactive protein; ESR—erythrocyte sedimentation rate; ATA—antitopoisomerase 1 antibodies; ACA—anticentromer antibodies; RNA III-pol—antiRNA III polymerase antibodies; mRSS—modified Rodnan skin score; NVC—nailfold capillaroscopy; ILD—interstitial lung disease; FVC—forced vital capacity; DLCO—diffusing lung capacity of carbon monoxide; 6MWD—six-minute walking distance; PH—pulmonary hypertension.

**Table 4:** Univariable logistic regression of MACE predictors.

Variable	OR	IC95%	*p*
Cardiovascular risk factor			
Age at diagnosis	1.03	1.01–1.05	0.004
Male sex	1.81	0.87–3.77	0.114
Smoking	1.29	0.72–2.3	0.395
Arterial hypertension	2.33	1.35–4	0.002
Diabetes mellitus	0.45	0.1–2.1	0.313
Dyslipidemia + treatment	3.19	1.73–5.87	<0.001
SSc-specific features			
dcSSc	1.99	1.15–3.42	0.013
mRSS	1.04	1–1.07	0.018
ATA	1.59	0.93–2.74	0.094
NVC-late pattern	2.15	1.25–3.69	0.005
Digital ulcers	3.71	1.94–7.07	<0.001
Scleroderma renal crisis	3.98	1.74–9.13	0.001
ILD	3.43	1.80–6.53	<0.001
FVC < 80	3.65	1.94–6.85	<0.001
DLCO < 75	2.27	1.21–4.24	0.01
PH	8.78	4.54–16.72	<0.001
NT-proBNP	1	1.000–1.001	0.018
CRP	1.05	1.02–1.08	<0.001
ESR	1.03	1.01–1.04	<0.001
Creatinine	1.31	1.04–1.65	0.024
Advanced vasodilators	3.2	1.8–5.68	<0.001

dcSSc—diffuse cutaneous involvement systemic sclerosis; CRP—C-reactive protein; ESR—erythrocyte sedimentation rate; ATA—antitopoisomerase 1 antibodies; mRSS—modified Rodnan skin score; NVC—nailfold capillaroscopy; ILD—interstitial lung disease; FVC—forced vital capacity; DLCO—diffusing lung capacity of carbon monoxide; PH—pulmonary hypertension.

**Table 5:** Multivariable logistic regression of MACE predictors—classic model.

Predictor	OR	IC95%	*p*
Age at diagnosis	1	0.98–1.04	0.517
Male sex	2.06	0.85–4.97	0.108
Smoking	1.26	0.62–2.57	0.52
Arterial hypertension	1.75	0.89–3.42	0.1
Diabetes mellitus	0.14	0.01–1.1	0.06
Dyslipidemia + treatment	2.72	1.37–5.4	0.004

**Table 6:** Comparison of the performance of the classical cardiovascular model and extended models incorporating systemic sclerosis-specific variables.

MACE Model	X^2^	*p*	Nagelkerke R^2^	AUC
Classic	26.2	<0.001	0.149	0.708
Classic + mRSS	29.6	<0.001	0.168	0.722
Classic + dcSSc	30.1	<0.001	0.172	0.721
Classic + ATA	30.6	<0.001	0.173	0.728
Classic + late NVC pattern	32.7	<0.001	0.184	0.731
Classic + ILD	35.6	<0.001	0.199	0.733
Classic + CRP	49.1	<0.001	0.267	0.760
Classic + PH	60.8	<0.001	0.326	0.811

mRSS—modified Rodnan skin score; dcSSc—diffuse cutaneous involvement systemic sclerosis; ATA—antitopoisomerase 1 antibodies; NVC—nailfold capillaroscopy; ILD—interstitial lung disease; CRP—C-reactive protein; PH—pulmonary hypertension.

**Table 7:** Multivariable analysis of the exploratory cardiovascular risk model.

Predictor	OR	IC95%	*p*
Dyslipidemia + treatment	4.26	1.99–9.13	<0.001
dcSSc	2.02	1.02–4.01	0.043
PH	8.39	3.88–18.13	<0.001
CRP	1.04	1.02–1.08	0.002

dcSSc—diffuse cutaneous involvement systemic sclerosis; CRP—C-reactive protein; PH—pulmonary hypertension.

## Data Availability

All the details are found in the Supplementary Materials.

## Supplementary Materials

The following supporting information can be downloaded at: https://www.mdpi.com/article/10.3390/diagnostics16193070/s1, Supplementary Materials: all the detailed data. References [27,28,29] are cited in the Supplementary Materials.

## Abbreviations

The following abbreviations are used in this manuscript:

SSc	systemic sclerosis
MACE	major adverse cardiovascular events
AUC	area under the curve
ROC	receiver operating characteristic
OR	odd ratio
CI	confidence interval
SD	standard deviation
n	number
IQR	interquartile range
dcSSc	diffuse cutaneous systemic sclerosis
lcSSc	limited cutaneous systemic sclerosis
LDL-low	density lipoprotein
HDL-high	density lipoprotein
CRP	C reactive protein
ESR	erythrocyte sedimentation rate
ATA	antitopoisomerase 1 antibodies
ACA	anticentromer antibodies
RNA III-pol	antiRNA III polymerase antibodies
mRSS	modified Rodnan skin score
ILD	interstitial lung disease
FVC	forced vital capacity
DLCO	diffusing lung capacity of carbon monoxide
6MWD	six-minute walking distance
PH	pulmonary hypertension
mPAP	mean pulmonary arterial pressure
NVC	nailfold videocapillaroscopy
