# Supplementary material for: Systemic Sclerosis-Specific Determinants of Major Adverse Cardiovascular Events: Development of an Exploratory Cardiovascular Risk Model Beyond Traditional Risk Factors
Source: Diagnostics (Basel). 2026 Sep 22;16(19):3070. doi: 10.3390/diagnostics16193070 (PMC13663982; doi:10.3390/diagnostics16193070)
Supplement: Supplementary file 1 [file diagnostics-16-03070-s001.zip › diagnostics-4523131-supplementary.pdf]

Results

Descriptives_continuous

Descriptives

	Col Tot	HDL	LDL	TG	Age at inclusion	Age at diagnosis	Per Urmarire (Luni)	Hb	O2saturationatrest	Baseline6WMT(m)	sPAP (mmHg)	DLCO 0 (%)	FVC 0 (%)	eRFG	creatinina	[illegible]
N	193	131	166	166	272	272	272	272	139	123	255	242	257	270	272	
Mean	188	54.1	102	101	53.3	50.4	59.2	12.6	96.7	445	31.2	63.7	83.1	66.4	1.18	
Median	187	52	98.0	91.0	54.0	51.0	36.0	13.0	97	450	28	67.0	84.0	66.7	0.910	
Standard deviation	45.7	18.7	38.2	54.5	11.8	12.8	56.7	2.01	2.12	132	14.8	19.2	19.8	21.2	1.12	
Minimum	72	10	31	31	24	10	2	0.00	86	112	10	0.00	31.0	4.00	0.520	
Maximum	325	116	216	539	85	85	276	17.3	100	789	124	119	139	139	10.1	[illegible]
Shapiro-Wilk W	0.995	0.951	0.981	0.711	0.992	0.996	0.764	0.847	0.846	0.991	0.739	0.978	0.991	0.967	0.375	[illegible]
Shapiro-Wilk p	.806	<.001	.026	<.001	.165	.716	<.001	<.001	<.001	.628	<.001	<.001	.112	<.001	<.001	[illegible]
25th percentile	154	42.0	71.8	70.0	44.0	41.0	24.0	11.8	96.0	361	22.0	52.0	70.0	55.9	0.810	
75th percentile	220	62.0	129	118	62.0	59.0	72.0	13.7	98.0	522	33.5	77.0	98.0	77.9	1.05	

Descriptives

Descriptives

	Dyspnea (NYHA)	DLCOless75	Pattern capil	Gender	Vital status	Smoker	HTA Y/N	Pericard	HF	Maj card inv	Aritmii(ECG)	DZ Y/N	Skin invol	Raynauds	DUs	dc or lc (dc=1)	ATA	ARNpol	CRP>5	[illegible]
N	265	264	272	272	272	272	268	262	270	267	272	260	272	272	254	267	271	271	272	[illegible]

Frequencies

Frequencies of Dyspnea (NYHA)

Dyspnea (NYHA)	Counts	% of Total	Cumulative %
0	15.0	5.7%	5.7%
1	44.0	16.6%	22.3%
2	112.0	42.3%	64.5%
3	80.0	30.2%	94.7%
4	14.0	5.3%	100.0%

Frequencies of DLCOless75

DLCOless75	Counts	% of Total	Cumulative %
1	175.0	66.3%	66.3%
0	89.0	33.7%	100.0%

Frequencies of Pattern capil

Pattern capil	Counts	% of Total	Cumulative %
0	11.0	4.0%	4.0%
1	38.0	14.0%	18.0%
2	120.0	44.1%	62.1%
3	103.0	37.9%	100.0%

Frequencies of Gender

Gender	Counts	% of Total	Cumulative %
0	236.0	86.8%	86.8%
1	36.0	13.2%	100.0%

Frequencies of Vital status

Vital status	Counts	% of Total	Cumulative %
0	234.0	86.0%	86.0%
1	38.0	14.0%	100.0%

Frequencies of Smoker

Smoker	Counts	% of Total	Cumulative %
0	197.0	72.4%	72.4%
1	75.0	27.6%	100.0%

Frequencies of HTA Y/N

HTA Y/N	Counts	% of Total	Cumulative %
0	149	55.6%	55.6%
1	119	44.4%	100.0%

Frequencies of Pericard

Pericard	Counts	% of Total	Cumulative %
0	228.0	87.0%	87.0%
1	34.0	13.0%	100.0%

Frequencies of HF

HF	Counts	% of Total	Cumulative %
0	191.0	70.7%	70.7%
1	79.0	29.3%	100.0%

Frequencies of Maj card inv

Maj card inv	Counts	% of Total	Cumulative %
0	170.0	63.7%	63.7%
1	97.0	36.3%	100.0%

Frequencies of Aritmii(ECG)

Aritmii(ECG)	Counts	% of Total	Cumulative %
0	169	62.1%	62.1%
1	103	37.9%	100.0%

Frequencies of DZ Y/N

DZ Y/N	Counts	% of Total	Cumulative %
0	247.0	95.0%	95.0%
1	13.0	5.0%	100.0%

Frequencies of Skin invol

Skin invol	Counts	% of Total	Cumulative %
0	95.0	34.9%	34.9%
1	177.0	65.1%	100.0%

Frequencies of Raynauds

Raynauds	Counts	% of Total	Cumulative %
0	23.0	8.5%	8.5%
1	249.0	91.5%	100.0%

Frequencies of DUs

DUs	Counts	% of Total	Cumulative %
0	114	44.9%	44.9%
1	140	55.1%	100.0%

Frequencies of dc or lc (dc=1)

dc or lc (dc=1)	Counts	% of Total	Cumulative %
0	133	49.8%	49.8%
1	134	50.2%	100.0%

Frequencies of ATA

ATA	Counts	% of Total	Cumulative %
0	119	43.9%	43.9%
1	152	56.1%	100.0%

Frequencies of ARNpol

ARNpol	Counts	% of Total	Cumulative %
0	259.0	95.6%	95.6%
1	12.0	4.4%	100.0%

Frequencies of CRP>5

CRP>5	Counts	% of Total	Cumulative %
0	131	48.2%	48.2%
1	141	51.8%	100.0%

Frequencies of Criza renala

Criza renala	Counts	% of Total	Cumulative %
0	246.0	90.4%	90.4%
1	26.0	9.6%	100.0%

Frequencies of Probable ILDyesno

Probable ILDyesno	Counts	% of Total	Cumulative %
0	82.0	30.1%	30.1%
1	190.0	69.9%	100.0%

Frequencies of Fibroza pulmonara

Fibroza pulmonara	Counts	% of Total	Cumulative %
0	97.0	35.7%	35.7%
1	175.0	64.3%	100.0%

Frequencies of FVC<80 ever

FVC<80 ever	Counts	% of Total	Cumulative %
0	126	53.2%	53.2%
1	111	46.8%	100.0%

Frequencies of HTP

HTP	Counts	% of Total	Cumulative %
0	212.0	78.8%	78.8%
1	57.0	21.2%	100.0%

Frequencies of CSt

CSt	Counts	% of Total	Cumulative %
0	153	56.3%	56.3%
1	119	43.8%	100.0%

Frequencies of IS

IS	Counts	% of Total	Cumulative %
0	44.0	16.2%	16.2%
1	228.0	83.8%	100.0%

Frequencies of IS Clasic Y/N

IS Clasic Y/N	Counts	% of Total	Cumulative %
0	47.0	17.3%	17.3%
1	225.0	82.7%	100.0%

Frequencies of IS Biologic Y/N

IS Biologic Y/N	Counts	% of Total	Cumulative %
0	248.0	92.2%	92.2%
1	21.0	7.8%	100.0%

Frequencies of VD Clasic YN

VD Clasic YN	Counts	% of Total	Cumulative %
0	69.0	25.4%	25.4%
1	203.0	74.6%	100.0%

Frequencies of VD Avansat Y/N

VD Avansat Y/N	Counts	% of Total	Cumulative %
0	198.0	72.8%	72.8%
1	74.0	27.2%	100.0%

Frequencies of ACA

ACA	Counts	% of Total	Cumulative %
0	177.0	65.3%	65.3%
1	94.0	34.7%	100.0%

Frequencies of Statine

Statine	Counts	% of Total	Cumulative %
0	113	44.7%	44.7%
1	140	55.3%	100.0%

Frequencies of Trat HTA

Trat HTA	Counts	% of Total	Cumulative %
0	149	55.8%	55.8%
1	118	44.2%	100.0%

Descriptives

Descriptives

	MACE_any	MACE_primaryY/N	MACE_extendedY/N	MACE_Type_First	CV_death	MACE_after_SSc
N	272	268	269	269	270	267

Frequencies

Frequencies of MACE_any

MACE_any	Counts	% of Total	Cumulative %
0	195.0	71.7%	71.7%
1	77.0	28.3%	100.0%

Frequencies of MACE_primaryY/N

MACE_primaryY/N	Counts	% of Total	Cumulative %
0	247.0	92.2%	92.2%
1	21.0	7.8%	100.0%

Frequencies of MACE_extendedY/N

MACE_extendedY/N	Counts	% of Total	Cumulative %
0	192.0	71.4%	71.4%
1	77.0	28.6%	100.0%

Frequencies of MACE_Type_First

MACE_Type_First	Counts	% of Total	Cumulative %
0	191.00	71.0%	71.0%
1	7.00	2.6%	73.6%
2	5.00	1.9%	75.5%
3	5.00	1.9%	77.3%
4	3.00	1.1%	78.4%
5	34.00	12.6%	91.1%
6	11.00	4.1%	95.2%
7	5.00	1.9%	97.0%
8	5.00	1.9%	98.9%
9	2.00	0.7%	99.6%
10	1.00	0.4%	100.0%

Frequencies of CV_death

CV_death	Counts	% of Total	Cumulative %
0	260.0	96.3%	96.3%
1	10.0	3.7%	100.0%

Frequencies of MACE_after_SSc

MACE_after_SSc	Counts	% of Total	Cumulative %
0	192.00	71.9%	71.9%
1	74.00	27.7%	99.6%
2026	1.00	0.4%	100.0%

Independent Samples T-Test

Independent Samples T-Test

		Statistic	df	p
Age at diagnosis	Student's t	-2.9982	267.0	.003
	Mann-Whitney U	5566		.002
Age at inclusion	Student's t	-3.0152	267.0	.003
	Mann-Whitney U	5502		.001
Disease duration(yr)	Student's t	-0.5382	267.0	.591
	Mann-Whitney U	6928		.420
Per Urmarire (Luni)	Student's t	-1.6630	267.0	.097
	Mann-Whitney U	6414		.088
BMI	Student's t	0.0645	266.0	.949
	Mann-Whitney U	7157		.732
Col Tot	Student's t	4.0291	190.0	<.001
	Mann-Whitney U	2393		<.001
HDL	Student's t	0.8542[a]	128.0	.395
	Mann-Whitney U	1552		.172
LDL	Student's t	2.8311	164.0	.005
	Mann-Whitney U	1983		.005
TG	Student's t	-0.9950	163.0	.321
	Mann-Whitney U	2728		.872
mRSS	Student's t	-2.4384	267.0	.015
	Mann-Whitney U	5770		.005
CRP	Student's t	-4.9200[a]	267.0	<.001
	Mann-Whitney U	4842		<.001
ESR	Student's t	-5.5147[a]	266.0	<.001
	Mann-Whitney U	4453		<.001
creatinina	Student's t	-2.5235[a]	267.0	.012
	Mann-Whitney U	5613		.002
eRFG	Student's t	3.4323[a]	265.0	<.001
	Mann-Whitney U	5214		<.001
FVC 0 (%)	Student's t	5.2706	252.0	<.001
	Mann-Whitney U	4110		<.001
DLCO 0 (%)	Student's t	4.8564	237.0	<.001
	Mann-Whitney U	3781		<.001
sPAP (mmHg)	Student's t	-6.9344[a]	250.0	<.001
	Mann-Whitney U	3673		<.001
Baseline6WMT(m)	Student's t	2.2613	118.0	.026
	Mann-Whitney U	1160		.033
O2saturationatrest	Student's t	2.1494	134.0	.033
	Mann-Whitney U	1441		.027
NT-proBNP	Student's t	-2.9746[a]	83.0	.004
	Mann-Whitney U	397		<.001

Note. H_a $\mu_0 \neq \mu_1$

[a] Levene's test is significant ($p < .05$), suggesting a violation of the assumption of equal variances

Group Descriptives

	Group	N	Mean	Median	SD	SE
Age at diagnosis	0	192	48.90	49.500	12.773	0.9218
	1	77	54.01	54.00	12.30	1.402
Age at inclusion	0	192	51.90	52.000	11.578	0.8356
	1	77	56.64	57.00	11.80	1.345
Disease duration(yr)	0	192	7.61	4.000	9.229	0.6660
	1	77	8.29	5.00	9.53	1.086
Per Urmarire (Luni)	0	192	56.03	36.000	54.000	3.8971
	1	77	68.73	48.00	62.73	7.148
BMI	0	191	22.38	22.000	3.841	0.2779
	1	77	22.35	22.50	3.46	0.395
Col Tot	0	139	195.23	197.000	43.282	3.6712
	1	53	166.60	163.00	45.89	6.303
HDL	0	89	54.98	53.000	16.410	1.7394
	1	41	51.95	47.00	23.13	3.612
LDL	0	120	106.88	103.000	37.583	3.4308
	1	46	88.52	84.50	36.91	5.442
TG	0	118	97.88	91.500	43.066	3.9646
	1	47	107.23	88.00	76.19	11.114
mRSS	0	192	9.25	7.000	7.172	0.5176
	1	77	11.71	8.00	8.24	0.939
CRP	0	192	5.37	3.000	7.384	0.5329
	1	77	25.13	6.00	54.57	6.218
ESR	0	191	20.17	14.000	17.695	1.2803
	1	77	36.09	33.00	28.61	3.261
creatinina	0	192	1.07	0.900	0.968	0.0698
	1	77	1.45	1.00	1.41	0.160
eRFG	0	191	69.22	69.700	18.601	1.3459
	1	76	59.48	59.85	25.87	2.968
FVC 0 (%)	0	179	87.22	89.000	18.563	1.3874
	1	75	73.53	75.00	19.65	2.268
DLCO 0 (%)	0	171	67.37	69.600	17.736	1.3563
	1	68	54.60	56.00	19.79	2.400
sPAP (mmHg)	0	175	27.33	26.000	9.081	0.6865
	1	77	40.26	34.00	20.56	2.343
Baseline6WMT(m)	0	83	464.00	463.000	125.252	13.7482
	1	37	405.32	391.00	144.02	23.677
O2saturationatrest	0	97	96.96	97.000	2.015	0.2046
	1	39	96.10	96.00	2.30	0.369
NT-proBNP	0	44	404.15	146.000	669.827	100.9802
	1	41	4965.33	863.00	10152.09	1585.490

Contingency Tables

Contingency Tables

		MACE_extendedY/N		
Capilaroscopic 3		0	1	Total
0	Observed	130	38	168
	% within column	67.7%	49.4%	62.5%
1	Observed	62	39	101
	% within column	32.3%	50.6%	37.5%
Total	Observed	192	77	269
	% within column	100.0%	100.0%	100.0%

χ^2 Tests

	Value	df	p
χ^2	7.90	1	.005
Fisher's exact test			.006
N	269		

Descriptives

Descriptives

	MACE_extendedY/N	Age at inclusion	Age at diagnosis	Per Urmarire (Luni)	Disease duration(yr)	BMI	Col Tot	HDL	LDL	TG	mRSS	CRP	ESR	NT-proBNP	CK	creatinina	eRFG
N	0	192	192	192	192	191	139	89	120	118	192	192	191	44	192	192	191
	1	77	77	77	77	77	53	41	46	47	77	77	77	41	77	77	76
Missing	0	0	0	0	0	1	53	103	72	74	0	0	1	148	0	0	1
	1	0	0	0	0	0	24	36	31	30	0	0	0	36	0	0	1
Mean	0	51.9	48.9	56.0	7.61	22.4	195	55.0	107	97.9	9.25	5.37	20.2	404	98.7	1.07	69.2
	1	56.6	54.0	68.7	8.29	22.3	167	52.0	88.5	107	11.7	25.1	36.1	4965	154	1.45	59.5
Median	0	52.0	49.5	36.0	4.00	22.0	197	53	103	91.5	7.00	3.00	14	146	80.0	0.900	69.7
	1	57	54	48	5	22.5	163	47	84.5	88	8	6	33	863	85	1.00	59.9
Standard deviation	0	11.6	12.8	54.0	9.23	3.84	43.3	16.4	37.6	43.1	7.17	7.38	17.7	670	89.3	0.968	18.6
	1	11.8	12.3	62.7	9.53	3.46	45.9	23.1	36.9	76.2	8.24	54.6	28.6	10152	229	1.41	25.9
25th percentile	0	42.0	40.0	24.0	2.00	20.0	164	45.0	82.8	68.5	5.00	1.00	8.50	86.6	54.0	0.800	59.3
	1	51.0	48.0	24.0	3.00	21.0	134	36.0	59.3	71.5	6.00	3.00	17.0	263	50.0	0.840	44.3
75th percentile	0	59.0	58.0	72.0	9.00	24.0	223	62.0	132	121	12.0	6.25	28.5	350	108	1.01	79.6
	1	65.0	63.0	96.0	11.0	24.0	201	63.0	111	111	14.0	21.0	46.0	2773	129	1.22	74.8

Binomial Logistic Regression

Model Fit Measures

	Overall Model Test		
Model	χ^2	df	p
1	21.2	4	<.001

Note. Models estimated using sample size of N=243

Model Coefficients - MACE_extendedY/N

						95% Confidence Interval	
Predictor	Estimate	SE	Z	p	Odds ratio	Lower	Upper
Intercept	-2.6058	0.7422	-3.51	<.001	0.0738	0.0172	0.316
Smoker	0.3766	0.3314	1.14	.256	1.4573	0.7611	2.790
DZ Y/N	-1.8709	1.0667	-1.75	.079	0.1540	0.0190	1.246
Statine	1.1220	0.3376	3.32	<.001	3.0709	1.5844	5.952
Age at inclusion	0.0163	0.0135	1.20	.229	1.0164	0.9898	1.044

Note. Estimates represent the log odds of "MACE_extendedY/N = 1" vs. "MACE_extendedY/N = 0"

Binomial Logistic Regression

Model Fit Measures

		Overall Model Test		
Model	R^2_N	χ^2	df	p
1	0.133	26.1	1	<.001

Note. Models estimated using sample size of N=268

Model Coefficients - MACE_extendedY/N

Predictor	Estimate	SE	Z	p	Odds ratio	95% Confidence Interval	
						Lower	Upper
Intercept	-1.7388	0.23075	-7.54	<.001	0.176	0.112	0.276
ESR	0.0312	0.00669	4.67	<.001	1.032	1.018	1.045

Note. Estimates represent the log odds of "MACE_extendedY/N = 1" vs. "MACE_extendedY/N = 0"

Binomial Logistic Regression

Model Fit Measures

Model	R^2_N	Overall Model Test		
		χ^2	df	p
1	0.219	22.8	6	<.001

Note. Models estimated using sample size of N=241

Model Coefficients - MACE_primaryY/N

Predictor	Estimate	SE	Z	p	Odds ratio	95% Confidence Interval	
						Lower	Upper
Intercept	-5.5864	1.5302	-3.65085	<.001	0.00375	1.87e-4	0.0752
Age at diagnosis	0.0153	0.0238	0.64206	.521	1.01542	0.969	1.0640
Gender	1.4923	0.7212	2.06936	.039	4.44747	1.082	18.2795
Smoker	-0.4848	0.6906	-0.70198	.483	0.61582	0.159	2.3840
HTA Y/N	-0.2156	0.5568	-0.38719	.699	0.80606	0.271	2.4007
DZ Y/N	-16.4401	1796.5945	-0.00915	.993	7.25e-8	0.000	Inf
Statine	2.9257	1.0701	2.73414	.006	18.64742	2.290	151.8653

Note. Estimates represent the log odds of "MACE_primaryY/N = 1" vs. "MACE_primaryY/N = 0"

Prediction

Predictive Measures

AUC
0.813

Note. The cut-off value is set to 0.5

ROC Curve

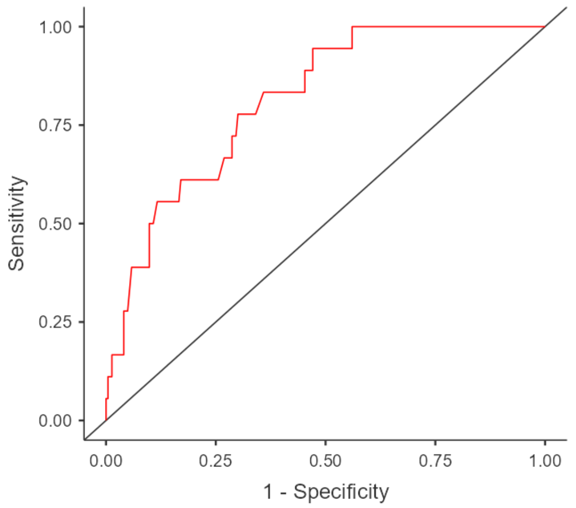

[3]

Pilot final

Model Fit Measures

		Overall Model Test		
Model	R^2_N	χ^2	df	p
1	0.281	32.5	4	<.001

Note. Models estimated using sample size of N=243

Model Coefficients - MACE_primaryY/N

						95% Confidence Interval	
Predictor	Estimate	SE	Z	p	Odds ratio	Lower	Upper
Intercept	-6.1621	1.36065	-4.53	<.001	0.00211	1.46e-4	0.0303
Statine	3.4874	1.29165	2.70	.007	32.70116	2.601	411.1686
dc or lc (dc=1)	0.8075	0.53331	1.51	.130	2.24222	0.788	6.3773
HTP	0.8555	0.53862	1.59	.112	2.35258	0.819	6.7611
CRP	0.0162	0.00734	2.21	.027	1.01637	1.002	1.0311

Note. Estimates represent the log odds of "MACE_primaryY/N = 1" vs. "MACE_primaryY/N = 0"

Prediction

Predictive Measures

AUC
0.813

Note. The cut-off value is set to 0.5

Binomial Logistic Regression

Model Fit Measures

		Overall Model Test		
Model	R^2_N	χ^2	df	p
1	0.239	24.9	7	<.001

Note. Models estimated using sample size of N=238

Model Coefficients - MACE_primaryY/N

						95% Confidence Interval	
Predictor	Estimate	SE	Z	p	Odds ratio	Lower	Upper
Intercept	-5.6712	1.5441	-3.6728	<.001	0.00344	1.67e-4	0.0710
Age at diagnosis	0.0151	0.0239	0.6331	.527	1.01526	0.969	1.0640
Gender	1.1781	0.7369	1.5987	.110	3.24815	0.766	13.7687
Smoker	-0.3898	0.6798	-0.5735	.566	0.67717	0.179	2.5664
HTA Y/N	-0.2951	0.5652	-0.5222	.602	0.74442	0.246	2.2539
Statine	2.8022	1.0681	2.6236	.009	16.48127	2.032	133.7037
DZ Y/N	-15.1603	1141.9656	-0.0133	.989	2.61e-7	0.000	Inf
HTP	0.9140	0.5557	1.6448	.100	2.49426	0.839	7.4121

Note. Estimates represent the log odds of "MACE_primaryY/N = 1" vs. "MACE_primaryY/N = 0"

Prediction

Predictive Measures

AUC
0.800

Note. The cut-off value is set to 0.5

ROC Curve

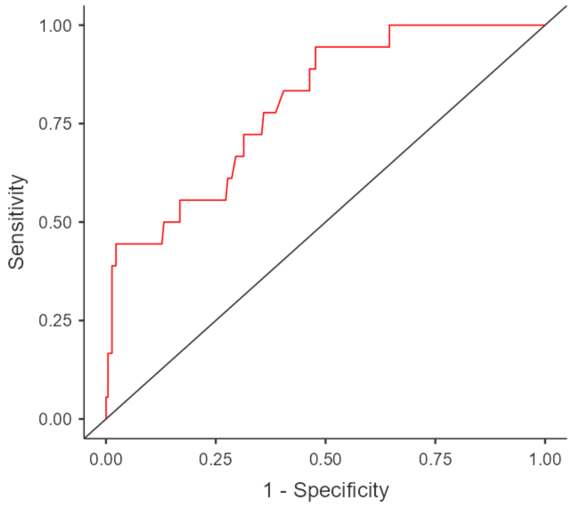

[3]

References

[1] The jamovi project (2025). *jamovi*. (Version 2.7) [Computer Software]. Retrieved from https://www.jamovi.org.

[2] R Core Team (2025). *R: A Language and environment for statistical computing*. (Version 4.5) [Computer software]. Retrieved from https://cran.r-project.org. (R packages retrieved from CRAN snapshot 2025-05-25).

[3] Sing, T., Sander, O., Beerenwinkel, N., & Lengauer, T., Unterthiner, T., & Ernst, F. G. M. (2020). *ROCR: Visualizing the Performance of Scoring Classifiers*. [R package]. Retrieved from https://cran.r-project.org/package=ROCR.
